# On the Use of Veratrum Viride in Fevers

**Published:** 1856-01

**Authors:** N. S. Davis, J. R. Mills


ARTICLE II. — *On the Use of Veratrum Viride in Fevers.* A Letter from Huntingdon, Indiana, addressed to one of the Editors.

DEAR SIR,—I hope it will not prove a source of annoyance to give you the result of my experience in the use of veratrum viride as a diaphoretic in the hot stage of our autumnal fevers.

I noticed with interest your observations in your Journal, sometime last spring, upon the use of this agent in puerperal peritonitis; and from your opinion of its modus operandi, it suggested itself to me, that it would be an excellent agent in the hot stage of fever, as above stated, and determined upon giving it a trial in the first favorable opportunity. In July last, when fevers became prevalent here, I gave it in teaspoonful doses to an adult, during the hot stage, with the result that far surpassed my expectation. It not only reduced the heart's action rapidly, but at the same time brought about free diaphoresis; its action was so certain in every case, that in my pill bags it soon occupied the space set apart for the pulvis ipecac. et opii.

I used it in the form you recommended, excepting water instead of the camphorated tinct. of opium, for no other object than to render its administration more certain, being easier telling when a teaspoon is full than when only two-thirds.

R. Sat. Tinct. Veratrum Viride, - iʒ
Spts. Nitre Dulcis, . - - i℥
Water, - - - - - - i℥

(I gave it indiscriminately in every temperament and age, and to the extent of producing free emesis, and found no bad effect when it was given to the extent of producing emesis; it also produced considerable prostration, but that, together with the vomiting, would cease spontaneously in one or two hours after withholding the medicine, at which time I would invariably find my patient in a profuse sweat, and in a fine condition for the administration of quinine, and, if given freely, had no return of fever.)

It would produce such profuse sweating and relaxation, that I sometimes thought it was a great pity Sam. Thompson had not known of it; it would have been such a saving to them in the way of fuel, expended for making steam. It acted so well in my hands, that I suggested to my friend, Dr. Greyston, of this place, to give it a trial also, which he did with as favorable a result. I will give briefly a few cases:

Master Hardman, aged 10, after a chill, had suffered three days, with a high grade of fever, with a very slight remission, if any, in spite of the Dover's powders he had taken every two hours, in proper doses, and *bitter* herb tea, as recommended by Dr. S. of this place; at this time I saw him, and found him talking incoherently; as you would expect, at this stage, skin dry and hot, tongue and mouth dry, &c. I gave him one-half of a teaspoonful of the mixture every hour, until he had taken seven doses, when he commenced vomiting freely, and continued to do so for about one and a-half hours, when it subsided, leaving him in the condition I have above stated, sweating freely, pulse reduced to about 90; I gave quinine freely, and he had no return of the fever.

The above case will answer in every particular for a number of other cases with the same result.

A case that I think is as much to the point, was the following: a Mrs. E. was attacked with a continued form of fever; I had got out of the saturated tincture; I had some more preparing at this time, and I was compelled to use the Dover's powder as a substitute, which I gave freely, alternating it with spts. nitre for three days; but the fever went on, regardless of my dosing. I remarked to the lady, that it seemed a difficult matter to get her into a sweating stage. "Oh!" said she, "Doctor, you must not wait to get me to sweat before you cure the fever; for if you do, you will let me die. For Dr. Leaper, of Ohio, tried ten days once, when I had the fever, to make me sweat, and could not, and had to cure me without it; I never sweat, Doctor, sick or well." I still continued my Dover's powders, with all the adjuncts I could bring to bear, for three days longer, when I had almost concluded with the Ohio Dr., to cure her without sweating. I thought, however, my tincture had some strength, and I gave it *ad libitum* until I produced free emesis—a very profuse sweating. I gave her four grains of quinine every three hours, without any return of the fever; and here it will not be out of place to say, that I have found no case of fever during the past summer, (and we have had a great many on the Wabash the past summer and fall,) that has not given way with this size doses of the quinine.

I submit the above for what they may be worth, only hoping that some of my medical brethren, whose discriminating powers are better than my own, will give it a further trial. I have used it in but one case of pneumonia; but I believe it will be a great adjuvant in the treatment of that disease.

Yours, respectfully,

Prof. N. S. Davis. J. R. Mills, M. D.

